# Association of Age, Antipsychotic Medication, and Symptom Severity in Schizophrenia With Proton Magnetic Resonance Spectroscopy Brain Glutamate Level: A Mega-analysis of Individual Participant-Level Data

**DOI:** 10.1001/jamapsychiatry.2021.0380

**Published:** 2021-04-21

**Authors:** Kate Merritt, Philip K. McGuire, Alice Egerton, and the 1H-MRS in Schizophrenia Investigators, André Aleman, Wolfgang Block, Oswald J. N. Bloemen, Faith Borgan, Juan R. Bustillo, Aristides A. Capizzano, Jennifer Marie Coughlin, Camilo De la Fuente-Sandoval, Arsime Demjaha, Kara Dempster, Kim Q. Do, Fei Du, Peter Falkai, Beata Galinska-Skok, Jurgen Gallinat, Charles Gasparovic, Cedric E. Ginestet, Naoki Goto, Ariel Graff-Guerrero, Beng Choon Ho, Oliver D. Howes, Sameer Jauhar, Peter Jeon, Tadafumi Kato, Charles A. Kaufmann, Lawrence S. Kegeles, Matcheri Keshavan, Sang-Young Kim, Hiroshi Kunugi, John Lauriello, Edith Jantine Liemburg, Meghan E. Mcilwain, Gemma Modinos, Elias D. Mouchlianitis, Jun Nakamura, Igor Nenadic, Dost Öngür, Miho Ota, Lena Palaniyappan, Christos Pantelis, Eric Plitman, Sotirios Posporelis, Scot E. Purdon, Jürgen R. Reichenbach, Perry F. Renshaw, Bruce R. Russell, Akira Sawa, Martin Schaefer, Dikoma C. Shungu, Stefan Smesny, Jeffrey A. Stanley, James M. Stone, Agata Szulc, Reggie Taylor, Katy Thakkar, Jean Théberge, Philip G. Tibbo, Therese van Amelsvoort, Jerzy Walecki, Peter C. Williamson, Stephen James Wood, Lijing Xin, Hidenori Yamasue

**Affiliations:** 1 Division of Psychiatry, Institute of Mental Health, UCL, London, United Kingdom; 2 Psychosis Studies Department, Institute of Psychiatry, Psychology, and Neuroscience, King’s College London, London, United Kingdom; 3 Center for Brain Disorder and Cognitive Science, Shenzhen University, Shenzhen, China; 4 University Medical Center Groningen, University of Groningen, Groningen, the Netherlands; 5 Department of Diagnostic and Interventional Radiology, University Hospital Bonn, Bonn, Germany; 6 Department of Psychiatry and Neuropsychology, Maastricht University, Maastricht, The Netherlands; 7 Department of Psychiatry and Behavioral Sciences, Center for Psychiatric Research, University of New Mexico School of Medicine, Albuquerque; 8 Department of Radiology, Division of Neuroradiology, University of Michigan, Ann Arbor; 9 Department of Psychiatry and Behavioral Sciences, Johns Hopkins University School of Medicine, Baltimore, Maryland; 10 Laboratory of Experimental Psychiatry, Instituto Nacional de Neurología y Neurocirugía, Mexico City, Mexico; 11 Neuropsychiatry Department, Instituto Nacional de Neurología y Neurocirugía, Mexico City, Mexico; 12 Department of Psychiatry, Dalhousie University, Halifax, Nova Scotia, Canada; 13 Center for Psychiatric Neuroscience, Department of Psychiatry, Lausanne University Hospital-CHUV, Prilly-Lausanne, Switzerland; 14 Psychotic Disorders Division, McLean Hospital, Harvard Medical School, Belmont, Massachusetts; 15 Department of Psychiatry, University Hospital, LMU Munich, Munich, Germany; 16 Department of Psychiatry, Medical University of Bialystok, Bialystok, Poland; 17 Department of Psychiatry and Psychotherapy, University Medical Center Hamburg-Eppendorf (UKE), Germany; 18 Mind Research Network, Albuquerque, New Mexico; 19 Department of Biostatistics and Health Informatics, Institute of Psychiatry, Psychology and Neuroscience King's College London, London, United Kingdom; 20 Department of Psychiatry, Kokura Gamo Hospital, Kitakyushu, Fukuoka, Japan; 21 Multimodal Neuroimaging Schizophrenia Group, Research Imaging Centre, Geriatric Mental Health Program at Centre for Addiction and Mental Health, Department of Psychiatry, University of Toronto, Toronto, Ontario, Canada; 22 Department of Psychiatry, University of Iowa Carver College of Medicine, Iowa City; 23 Department of Medical Biophysics, University of Western Ontario, London, Ontario, Canada; 24 Department of Psychiatry and Behavioral Science, Juntendo University Graduate School of Medicine, Tokyo, Japan; 25 Department of Psychiatry, Columbia University, New York State Psychiatric Institute, New York; 26 Harvard Medical School, Boston, Massachusetts; 27 Philips Healthcare, Seoul, Republic of Korea; 28 National Center of Neurology and Psychiatry, Kodaira, Tokyo, Japan; 29 Jefferson Health-Sidney Kimmel Medical College, Philadelphia, Pennsylvania; 30 Rob Giel Research Center, Department of Psychiatry, University Medical Center Groningen, Groningen, The Netherlands; 31 School of Pharmacy, University of Auckland, Grafton, Auckland, New Zealand; 32 Department of Neuroimaging, Centre for Neuroimaging Sciences, Institute of Psychiatry, Psychology & Neuroscience, De Crespigny Park, London, United Kingdom; 33 Department of Psychiatry, University of Occupational and Environmental Health, Kitakyushu, Fukuoka, Japan; 34 Editor, *JAMA Psychiatry*; 35 Department of Psychiatry, Western University, London, Ontario, Canada; 36 Melbourne Neuropsychiatry Centre, The University of Melbourne and Melbourne Health, Carlton, Victoria, Australia; 37 The Florey Institute of Neuroscience and Mental Health, Parkville, Victoria, Australia; 38 Cerebral Imaging Centre, Douglas Mental Health University Institute, Montreal, Quebec, Canada; 39 Department of Psychiatry, McGill University, Montreal, Quebec, Canada.; 40 South London and Maudsley, Bethlem Royal Hospital, Beckenham, United Kingdom; 41 Neuropsychology Department, Alberta Hospital Edmonton, Edmonton, Alberta, Canada; 42 Edmonton Early Intervention in Psychosis Clinic, Edmonton, Alberta, Canada; 43 Medical Physics Group, Institute for Diagnostic and Interventional Radiology, Jena University Hospital, Jena, Germany; 44 Department of Psychiatry, University of Utah, Salt Lake City; 45 School of Pharmacy, University of Otago, Dunedin, New Zealand; 46 Department of Psychiatry, Johns Hopkins University, Baltimore, Maryland; 47 Department of Neuroscience, Johns Hopkins University, Baltimore, Maryland; 48 Department of Mental Health, Johns Hopkins University, Baltimore, Maryland; 49 Department of Biomedical Engineering, Johns Hopkins University, Baltimore, Maryland; 50 Department of Genetic Medicine, Johns Hopkins University, Baltimore, Maryland; 51 Department of Psychiatry, Psychotherapy, Psychosomatics and Addiction Medicine, Kliniken Essen-Mitte, Essen, Germany; 52 Department of Psychiatry and Psychotherapy, Charité-Universitätsmedizin Berlin, Campus Charité Mitte, Berlin, Germany; 53 Department of Radiology, Weill Cornell Medical College, New York, New York; 54 Department of Psychiatry and Psychotherapy, Jena University Hospital, Jena, Germany; 55 Brain Imaging Research Division, Department of Psychiatry and Behavioral Neurosciences, Wayne State University School of Medicine, Detroit, Michigan; 56 Brighton and Sussex Medical School, University of Sussex, Brighton, United Kingdom; 57 Department of Psychiatry, Medical University of Warsaw, Poland; 58 Lawson Health Research Institute, London, Ontario, Canada; 59 Department of Psychology, Michigan State University, East Lansing; 60 Division of Psychiatry and Behavioral Medicine, Michigan State University, East Lansing; 61 Department of Psychiatry, Dalhousie University, Halifax, Nova Scotia, Canada; 62 Postgraduate Medical School, Warsaw, Poland; 63 Orygen, Melbourne, Australia; 64 Institute for Mental Health, University of Birmingham, Edgbaston, United Kingdom; 65 Centre for Youth Mental Health, University of Melbourne, Australia; 66 Animal Imaging and Technology Core, Center for Biomedical Imaging, Ecole Polytechnique Fédérale de Lausanne, Lausanne, Switzerland; 67 Department of Psychiatry, Hamamatsu University School of Medicine, Hamamatsu, Japan

## Abstract

**Importance:**

Proton magnetic resonance spectroscopy (1H-MRS) studies indicate that altered brain glutamatergic function may be associated with the pathophysiology of schizophrenia and the response to antipsychotic treatment. However, the association of altered glutamatergic function with clinical and demographic factors is unclear.

**Objective:**

To assess the associations of age, symptom severity, level of functioning, and antipsychotic treatment with brain glutamatergic metabolites.

**Data Sources:**

The MEDLINE database was searched to identify journal articles published between January 1, 1980, and June 3, 2020, using the following search terms: MRS or magnetic resonance spectroscopy and (1) schizophrenia or (2) psychosis or (3) UHR or (4) ARMS or (5) ultra-high risk or (6) clinical high risk or (7) genetic high risk or (8) prodrome* or (9) schizoaffective. Authors of 114 1H-MRS studies measuring glutamate (Glu) levels in patients with schizophrenia were contacted between January 2014 and June 2020 and asked to provide individual participant data.

**Study Selection:**

In total, 45 1H-MRS studies contributed data.

**Data Extraction and Synthesis:**

Associations of Glu, Glu plus glutamine (Glx), or total creatine plus phosphocreatine levels with age, antipsychotic medication dose, symptom severity, and functioning were assessed using linear mixed models, with study as a random factor.

**Main Outcomes and Measures:**

Glu, Glx, and Cr values in the medial frontal cortex (MFC) and medial temporal lobe (MTL).

**Results:**

In total, 42 studies were included, with data for 1251 patients with schizophrenia (mean [SD] age, 30.3 [10.4] years) and 1197 healthy volunteers (mean [SD] age, 27.5 [8.8] years). The MFC Glu (*F*_1,1211.9_ = 4.311, *P* = .04) and Glx (*F*_1,1079.2_ = 5.287, *P* = .02) levels were lower in patients than in healthy volunteers, and although creatine levels appeared lower in patients, the difference was not significant (*F*_1,1395.9_ = 3.622, *P* = .06). In both patients and volunteers, the MFC Glu level was negatively associated with age (Glu to Cr ratio, *F*_1,1522.4_ = 47.533, *P* < .001; cerebrospinal fluid–corrected Glu, *F*_1,1216.7_ = 5.610, *P* = .02), showing a 0.2-unit reduction per decade. In patients, antipsychotic dose (in chlorpromazine equivalents) was negatively associated with MFC Glu (estimate, 0.10 reduction per 100 mg; SE, 0.03) and MFC Glx (estimate, −0.11; SE, 0.04) levels. The MFC Glu to Cr ratio was positively associated with total symptom severity (estimate, 0.01 per 10 points; SE, 0.005) and positive symptom severity (estimate, 0.04; SE, 0.02) and was negatively associated with level of global functioning (estimate, 0.04; SE, 0.01). In the MTL, the Glx to Cr ratio was positively associated with total symptom severity (estimate, 0.06; SE, 0.03), negative symptoms (estimate, 0.2; SE, 0.07), and worse Clinical Global Impression score (estimate, 0.2 per point; SE, 0.06). The MFC creatine level increased with age (estimate, 0.2; SE, 0.05) but was not associated with either symptom severity or antipsychotic medication dose.

**Conclusions and Relevance:**

Findings from this mega-analysis suggest that lower brain Glu levels in patients with schizophrenia may be associated with antipsychotic medication exposure rather than with greater age-related decline. Higher brain Glu levels may act as a biomarker of illness severity in schizophrenia.

## Key Points

**Question:**

Are clinical and demographic factors associated with brain glutamate or glutamate plus glutamine (Glx) levels in schizophrenia?

**Findings:**

In this mega-analysis of 1251 patients with schizophrenia and 1197 healthy volunteers, medial frontal cortex glutamatergic metabolite levels were lower in patients and negatively associated with the dose of antipsychotic medication, although a reduction in glutamate levels with age was not accelerated in patients with schizophrenia compared with healthy individuals. Higher medial frontal cortex and medial temporal lobe glutamate levels were associated with more severe symptoms in patients with schizophrenia.

**Meaning:**

Lower brain glutamate levels may be associated with antipsychotic exposure rather than with greater age-related decline, whereas higher glutamate levels may serve as a biomarker of illness severity in patients with schizophrenia.

## Teaser

This mega-analysis assesses whether age, symptom severity, level of functioning, and antipsychotic treatment are associated with glutamate or glutamatergic metabolite levels measured with proton magnetic resonance spectroscopy in the medial frontal cortex or medial temporal lobe of patients with schizophrenia.

## Introduction

Glutamatergic dysfunction is implicated in the pathophysiology of schizophrenia,^1,2^ but the nature of this dysfunction may change over the course of illness.^2,3^ Aspects of glutamatergic dysfunction can be investigated in vivo using proton magnetic resonance spectroscopy (1H-MRS), which measures the total amount of intracellular and extracellular glutamate (Glu) in a predefined voxel of interest. Meta-analyses of 1H-MRS studies indicate that glutamatergic metabolites are elevated in patients with schizophrenia compared with healthy volunteers^2^; however, a recent meta-analysis of 7-T MRS studies reports lower Glu levels in patients,^4^ and individual studies show variable results. This heterogeneity may be associated with factors such as age, illness duration, symptom severity, illicit substance use, and antipsychotic medication exposure, which vary between cohorts. The associations of such factors are best examined in large data sets incorporating patients across different stages of illness.

There is some indication that elevations in 1H-MRS glutamatergic metabolite levels may be most apparent in early psychosis^5,6,7,8^ but reduced in chronic schizophrenia.^9,10,11,12,13,14^ This finding may be associated with the expression of dysfunctional compensatory processes that emerge secondary to the illness^15^ but may also be associated with other factors (eg, divergence from normal aging processes or medication exposure lasting many years). Large studies have not yet reached a consensus on the associations of aging with Glu levels in patients with schizophrenia. An age-related decrease in medial frontal cortex (MFC) Glu level has been observed in both patients and healthy volunteers,^16^ but these findings were not replicated by another large study.^17^ Alternatively, metaregression analysis has detected accelerated MFC glutamatergic reductions in patients with schizophrenia compared with healthy volunteers,^18,19,20^ but this finding was not apparent in a more recent analysis.^2^ Metaregression analyses are limited to using group mean data extracted from individual studies, and thus it is difficult to disentangle age-dependent associations from other clinical factors that correlate with age, such as the duration of illness or the duration of antipsychotic treatment. Indeed, a number of longitudinal studies have reported reductions in brain glutamatergic metabolite levels following antipsychotic treatment in the frontal and temporal lobes among other regions.^21^

There is also a lack of consensus about whether brain glutamatergic metabolite levels are associated with symptom severity and global functioning. A systematic review found inconsistent evidence to correlate Glu levels to symptom severity,^22^ although many studies were limited by small sample sizes of patients with similar symptom profiles. Individual studies comparing symptomatic and nonsymptomatic patients have reported higher Glu plus glutamine (Glx) levels in the symptomatic group^23^ and elevated Glu levels in nonremitted patients compared with remitted patients.^24,25,26^ However, age may confound these associations if patients with more severe symptoms are younger.

With the aim of better characterizing glutamatergic dysfunction in schizophrenia, we conducted a mega-analysis of individual participant–level data examining the associations of age, antipsychotic medication exposure, diagnosis, symptom severity, and functioning with 1H-MRS measures of glutamatergic metabolite levels. We hypothesized that (1) glutamatergic metabolite levels would decrease in association with age in both healthy volunteers and patients; (2) glutamatergic metabolite levels would be associated with a decrease in the context of higher antipsychotic medication doses; (3) glutamatergic metabolite levels would be lower in patients than in healthy volunteers; and (4) more severe symptoms and worse global functioning would be associated with higher Glu levels. In addition, we tested the assumption that these factors are not associated with the combined creatine and phosphocreatine signal (Cr) because Glu is commonly reported in ratio to Cr for analyses.

## Methods

The MEDLINE database was searched to identify journal articles published between January 1, 1980, and June 3, 2020, using the following search terms: MRS or magnetic resonance spectroscopy and (1) schizophrenia or (2) psychosis or (3) UHR or (4) ARMS or (5) ultra-high risk or (6) clinical high risk or (7) genetic high risk or (8) prodrome* or (9) schizoaffective. Authors of 1H-MRS studies were contacted at least twice between January 2014 and June 2020 to request anonymized participant-level 1H-MRS metabolite data, which included levels of Glu, glutamine, Glx, and Cr and Cramér-Rao Lower Bound values, which estimate metabolite goodness of fit.^27^ Clinical and demographic data included positive, negative, general, and total subscores of the Positive and Negative Syndrome Scale (PANSS), Global Assessment of Functioning (GAF) scores, Clinical Global Impression (CGI) scores, age, duration of illness, antipsychotic medication dose, and duration of treatment. All methods and results are reported following the Preferred Reporting Items for Systematic Reviews and Meta-analyses (PRISMA) reporting guideline.

Analyses were restricted to variables for which a minimum of 3 independent data sets were available.^28^ Brain metabolite data were categorized into (1) the MFC, including the anterior cingulate cortex and (2) medial temporal lobe (MTL), including the hippocampus. Data with Cramér-Rao Lower Bound values higher than 20% were excluded. Glutamate values are often corrected for the amount of cerebrospinal fluid (CSF) in the voxel because CSF contains negligible metabolites of interest. Alternatively, Glu is estimated relative to Cr. Herein, CSF-corrected and Cr-scaled values were aggregated separately.

Associations of Glu, Glx, or creatine levels with variables of interest were assessed using linear mixed models in R, version 3.6 (R Core Team),^29^ with the lmer and ggplot2 packages.^30^ Independent variables were entered as fixed factors, and study was entered as a random factor. Glutamate and Glx measures are not independent and thus were not corrected for multiple comparisons. Tests of collinearity determined which variables to include in the model. For analyses investigating the association of age with metabolites, model 1 investigated the direct association of age plus group (patient vs healthy volunteers), and model 2 assessed their interaction. The linear mixed models were estimated with maximum likelihoods because residual likelihoods are not comparable across models with different fixed effects.^31^ The lowest Akaike information criterion value indicates the best model,^28^ and χ^2^ tests were used to assess which model was superior. To determine whether metabolite values in patients were best estimated by age or with chlorpromazine equivalent (CPZE) dose (when both were significantly associated with metabolite levels), model 1 included CPZE dose, and model 2 included both age and CPZE dose.

For symptom severity, we reduced the number of comparisons by first testing the association between metabolite levels and PANSS total score. When the association was significant, follow-up analyses investigated the PANSS positive and negative score in one model with restricted maximum likelihoods.^28^ To investigate the association between Glu level and functioning, GAF scores were examined. If GAF scores were unavailable, then CGI scores were examined. To determine whether patient metabolite values were best estimated by age or by PANSS total scores, model 1 included PANSS total scores, and model 2 included both age and PANSS total scores. For all comparisons, a 2-sided *P* < .05 was considered statistically significant.

## Results

The literature search identified 114 studies (eFigure in the Supplement). Of those studies, 45 contributed data (eTable 1 in the Supplement). Two of these studies were not included because data were only available for ultra-high-risk participants,^32,33^ and 1 study was excluded because 1H-MRS was conducted using a J-resolved acquisition approach.^34^ A total sample size of 1251 patients with schizophrenia (mean [SD] age, 30.3 [10.4] years) and 1197 healthy volunteers (mean [SD] age, 27.5 [8.8] years) were included in the analyses. Sample sizes from each study ranged from 10 to 89 healthy volunteers and from 10 to 147 patients. Twenty-four studies examined patients with first-episode psychosis,^5,7,11,24,35,36,37,38,39,40,41,42,43,44,45,46,47,48,49,50,51,52,53^ and 20 studies examined patients with established schizophrenia.^11,14,25,35,36,38,39,40,44,54,55,56,57,58,59,60,61,62,63^ Four studies did not include healthy volunteer data.^24,32,61,64^

### Association of Demographic and Clinical Factors With Cr Level

**Figure 1.**
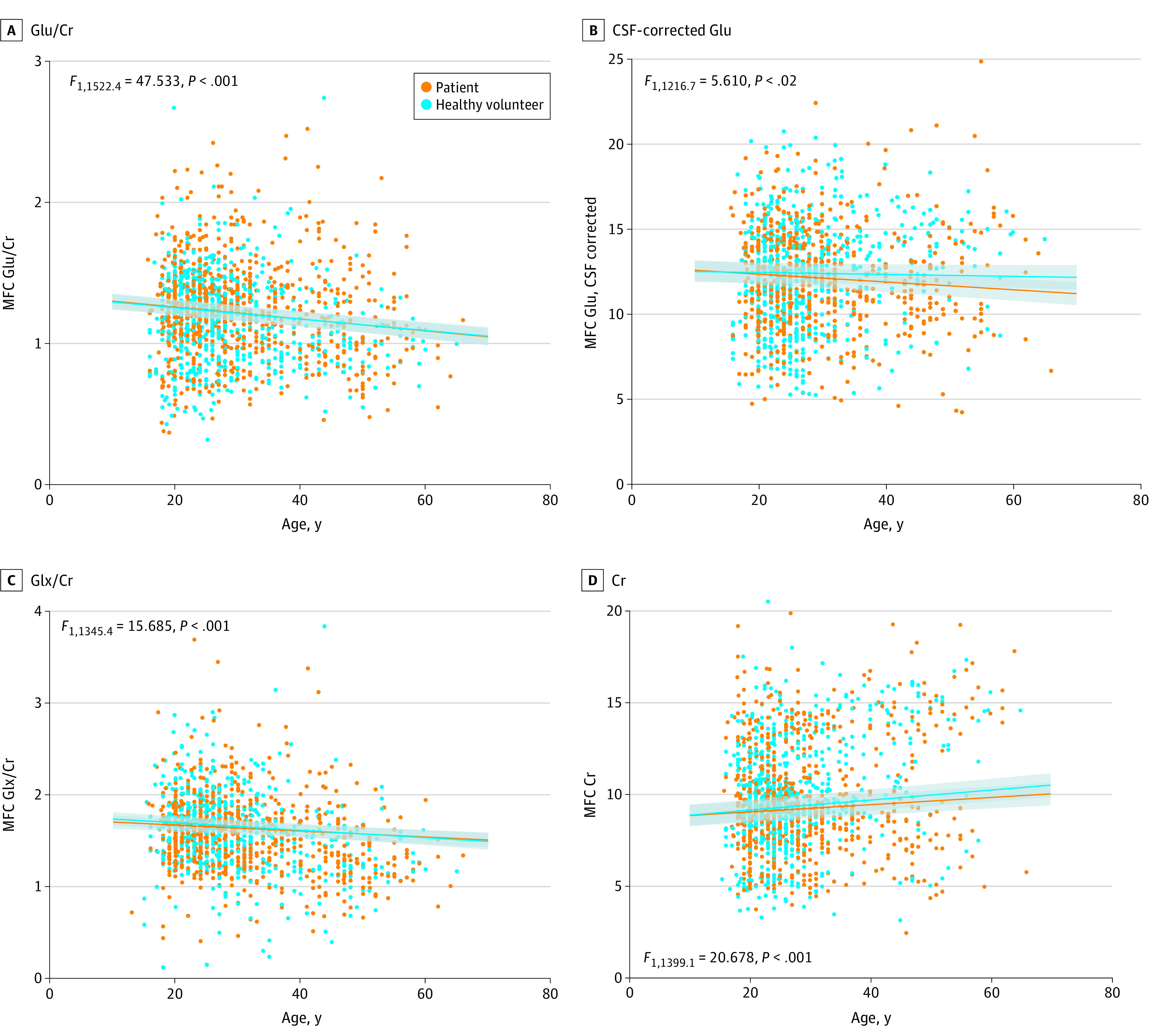
Medial Frontal Cortex (MFC) Glutamatergic Metabolite Levels by Age in Patients and in Healthy Volunteers A, MFC glutamate to creatinine plus phosphocreatine ratio (Glu/Cr). B, Cerebrospinal fluid (CSF)–corrected Glu levels. C, MFC Glu plus glutamine to Cr ratio (Glx/Cr). D, Cr levels. Glu and Glx levels in the MFC decrease with age in patients and healthy volunteers. Cr levels in the MFC increase with age in patients and healthy volunteers. Lines represent the linear mixed model for patients and healthy volunteers, with SEs represented by the gray shaded areas. The CSF-corrected Glu levels and the Cr levels are in arbitrary units.

In the MFC, Cr levels increased with age (*F*_1,1399.1_ = 20.678, *P* < .001; n = 1417) (Figure 1) at a rate of 0.2 units per decade (SE = 0.05). This association did not differ between patients and healthy volunteers (Table 1). In the MTL, there was no association between Cr level and age. There were no significant associations of Cr level with CPZE dose, PANSS total symptoms, or GAF score in either the MFC or MTL.

**Table 1.** Association of Age and Antipsychotic Medication With Glutamatergic Metabolite Levels and Total Creatine Plus Phosphocreatine Levels in Patients and Healthy Volunteers^a^

Brain region and source	P or HV, No.	Metabolite, estimated mean (SE)	Clinical variable, mean (SD)	Model 1: main effects	Model 2: interaction effects (age × diagnosis or CPZE dose + age)	Model comparison			
						AIC	Residual deviance	*P* value^b^	Model selected
Medial frontal cortex									
Cr									
24 Studies^5,7,11^ ^14,24,35,36,37,38^ ^40,42,43,44,45,46,47,50^ ^51,54,65,66,67,68,69^	P: 705 HV: 712	P: 9.20 (0.57) HV: 9.37 (0.57)	Age P: 29.55 (10.26) HV: 27.54 (8.62)	Age *F*_1,1399.1_ = 20.678, *P* < .001 Est (SE), 0.0227 (0.0050) Diagnosis *F*_1,1395.9_ = 3.622, *P* = .06	Age *F*_1,1399.4_ = 21.442, *P* < .001 Est (SE), 0.0197 (0.0061) Diagnosis *F*_1,1395.1_ = 0.042, *P* = .84 Age × diagnosis *F*_1,1394.1_ = 0.754, *P* = .38	Model 1: 5314.0 Model 2: 5315.3	Model 1: 5304.0 Model 2: 5303.3	.38	1
11 Studies^7,11,14^ ^24,37,38^ ^40,42,44,45,65^	P: 283	9.43 (0.68)	CPZE 364.14 (367.76)	CPZE *F*_1,273.8_ = 2.179, *P* = .14	NA				
Glutamate, Cr-scaled									
25 Studies^5,7,11^ ^14,24,25,35^ ^37,38,40,42,43,44,45,46,47^ ^50,51,57,59,60^ ^65,66,68,69^	P: 797 HV: 737	P: 1.21 (0.05) HV: 1.21 (0.05)	Age P: 30.53 (10.61) HV: 28.14 (8.85)	Age *F*_1,1522.4_ = 47.533, *P* < .001 Est (SE), −0.0042 (0.0006) Diagnosis *F*_1,1514.8_ = 0.071, *P* = .79	Age *F*_1,1522.3_ = 45.030, *P* < .001 Est (SE), −0.0042 (0.0007) Diagnosis *F*_1,1512.2_ = 0.060, *P* = .81 Age × diagnosis *F*_1,1511.2_ = 0.028, *P* = .87	Model 1: −577.18 Model 2: −575.21	Model 1: −550.5 Model 2: −543.2	.87	1
13 Studies ^7,11,14,24,37,38^ ^40,42,44,45,57,60,65^	P: 348	1.27 (0.08)	CPZE 394.10 (369.42)	CPZE *F*_1,338.5_ = 0.154, *P* = .70	NA				
Glutamate, CSF-corrected									
18 Studies^5,7,11^ ^14,24,35,38,40^ ^42,43,44,45,46,47,50,65,66,69^	P: 630 HV: 596	P: 12.10 (0.59) HV: 12.40 (0.59)	Age P: 29.87 (10.33) HV: 28.18 (8.81)	Age *F*_1,1216.7_ = 5.610, *P* = .02 Est (SE), −0.0161 (0.0068) Diagnosis *F*_1,1211.9_ = 4.311, *P* = .04 Est (SE), 0.2522 (0.1215)	Age *F*_1,1216.8_ = 4.241, *P* = .04 Est (SE) −0.0229 (0.0083) Diagnosis *F*_1,1209.9_ = 0.441, *P* = .51 Age × diagnosis *F*_1,1208.9_ = 2.001, *P* = .16	Model 1: 5243.5 Model 2: 5243.5	Model 1: 5233.5 Model 2: 5231.5	.16	1
10 Studies^7,11,14^ ^24,38,40,42,44,45,65^	P: 276	12.00 (0.70)	CPZE 382.09 (393.57)	CPZE *F*_1,269.3_ = 7.583, *P* = .006 Est (SE), −0.0010 (0.0003)	Age *F*_1,274.3_ = 17.109,<*P* = .001 Est (SE), −0.0574 (0.0139) CPZE *F*_1,269.4_ = 7.141, *P* = .008 Est (SE), −0.0009 (0.0003)	Model 1: 1223.3 Model 2: 1208.7	Model 1: 1215.3 Model 2: 1198.7	<.001	2
Glx, Cr-scaled									
24 Studies^5,7,11^ ^14,24,35,36,37,38,40^ ^42,43,44,46,47,49^ ^50,54,57,59,65,66,67,68^	P: 705 HV: 652	P: 1.65 (0.07) HV: 1.66 (0.07)	Age P: 30.74 (10.47) HV: 28.71 (9.04)	Age *F*_1,1345.4_ = 15.685, *P* < .001 Est (SE), −0.0036 (0.0009) Diagnosis *F*_1,1340.0_ = 1.036, *P* = .31	Age *F*_1,1346.2_ = 15.842, *P* < .001 Est (SE), −0.0033 (0.0011) Diagnosis *F*_1,1339.3_ = 0.650, *P* = .42 Age × diagnosis *F*_1,1336.6_ = 0.256, *P* = .61	Model 1: 543.84 Model 2: 545.58	Model 1: 533.84 Model 2: 533.58	.61	1
12 Studies^7,11,14^ ^24,37,38,40,42^ ^44,49,57,65^	P: 324	1.61 (0.11)	CPZE 400.64 (394.52)	CPZE *F*_1,314.9_ = 2.133, *P* = .14	NA				
Glx, CSF-corrected									
16 Studies^5,7,11^ ^14,24,35^ ^38,40,42,43,44^ ^46,47,50,65,66^	P: 573 HV: 519	P: 16.85 (1.04) HV: 17.31 (1.04)	Age P: 30.48 (10.57) HV: 28.78 (9.02)	Age *F*_1,1082.0_ = 0.631, *P* = .43 Diagnosis *F*_1,1079.2_ = 5.287, *P* = .02 Est (SE), 0.4574 (0.1989)	Age *F*_1,1082.5_ = 0.472, *P* = .49 Diagnosis *F*_1,1077.6_ = 1.321, *P* = .25 Age × diagnosis *F*_1,1076.8_ = 0.181, *P* = .67	Model 1: 5615.3 Model 2: 5617.1	Model 1: 5605.3 Model 2: 5605.1	.67	1
9 Studies^7,11,14^ ^24,38,40,42,44,65^	P: 259	15.80 (1.45)	CPZE 400.48 (424.59)	CPZE *F*_1251.3_ = 6.326, *P* = .01 Est (SE), −0.0011 (0.0004)	NA				
Medial temporal lobe									
Cr									
7 Studies^6,40,43^ ^52,53,67,68^	P: 120 HV: 157	P: 5.15 (1.09) HV: 5.13 (1.09)	Age P: 23.81 (5.75) HV: 24.40 (6.01)	Age *F*_1,270.5_ = 1.738, *P* = .19 Diagnosis *F*_1,270.6_ = 0.033, *P* = .86	Age *F*_1,270.5_ = 1.755, *P* = .19 Diagnosis *F*_1,270.3_ = 0.036, *P* = .85 Age × diagnosis *F*_1,270.2_ = 0.022, *P* = .88	Model 1: 731.91 Model 2: 733.89	Model 1: 721.91 Model 2: 721.89	.88	1
3 Studies^40,52,53^	P: 68	4.78 (2.83)	CPZE 237.49 (157.83)	*F*_1,65.1_ = 1.278, *P* = .26	NA				
Glx, Cr-scaled									
8 Studies^6,40,43^ ^52,53,56,67,70^	P: 143 HV: 151	P: 1.90 (0.14) HV: 1.91 (0.13)	Age P: 25.70 (6.93) HV: 24.20 (5.16)	Age *F*_1,293.7_ = 2.650, *P* = .10 Diagnosis *F*_1,293.9_ = 0.041, *P* = .84	Age *F*_1,293.7_ = 2.074, *P* = .15 Diagnosis *F*_1,290.6_ = 0.396, *P* = .53 Age × diagnosis *F*_1288.9_ = 0.494, *P* = .48	Model 1: 352.46 Model 2: 353.96	Model 1: 342.46 Model 2: 341.96	0.48	1
4 Studes^40,52^ ^53,56^	P: 94	1.93 (0.25)	CPZE 241.16 (154.68)	*F*_1,92.4_ = 0.450, *P* = .50	NA				

Abbreviations: AIC, Akaike information criterion; CPZE, chlorpromazine equivalent dose; Cr, creatine plus phosphocreatine; CSF, cerebrospinal fluid; Est, estimate; Glx, glutamate plus glutamine; HV, healthy volunteers; NA, not applicable; P, patients.

^a^ If age and CPZE dose are significantly associated with glutamatergic metabolites, then model 1 including CPZE is compared with model 2 including both CPZE and age. When χ^2^ test for model comparison is not significant, then the simplest model is selected.

^b^ Determined by use of the χ^2^ test.

### Association of Age and CPZE Dose With Glutamatergic Metabolite Levels

Duration of illness was associated with age and therefore was not included in the model. Age was not significantly associated with CPZE dose (eTable 2 in the Supplement).

Across all participants, MFC Glu levels decreased with age (Glu to Cr ratio: *F*_1,1522.4_ = 47.533, *P* < .001; n = 1534; CSF-corrected Glu level: *F*_1,1216.7_ = 5.610, *P* = .02; n = 1226) (Figure 1). The Glu to Cr ratio decreased by 0.04 units per decade (SE = 0.006), and CSF-corrected Glu levels decreased by 0.2 units per decade (SE = 0.07). There was no interaction between age and group (Table 1). The MFC Glx to Cr ratio also decreased with age (*F*_1,1345.4_ = 15.685, *P* < .001; n = 1357), by 0.04 units per decade (SE = 0.01). The MFC CSF-corrected Glx level was not significantly associated with age.

**Figure 2.**
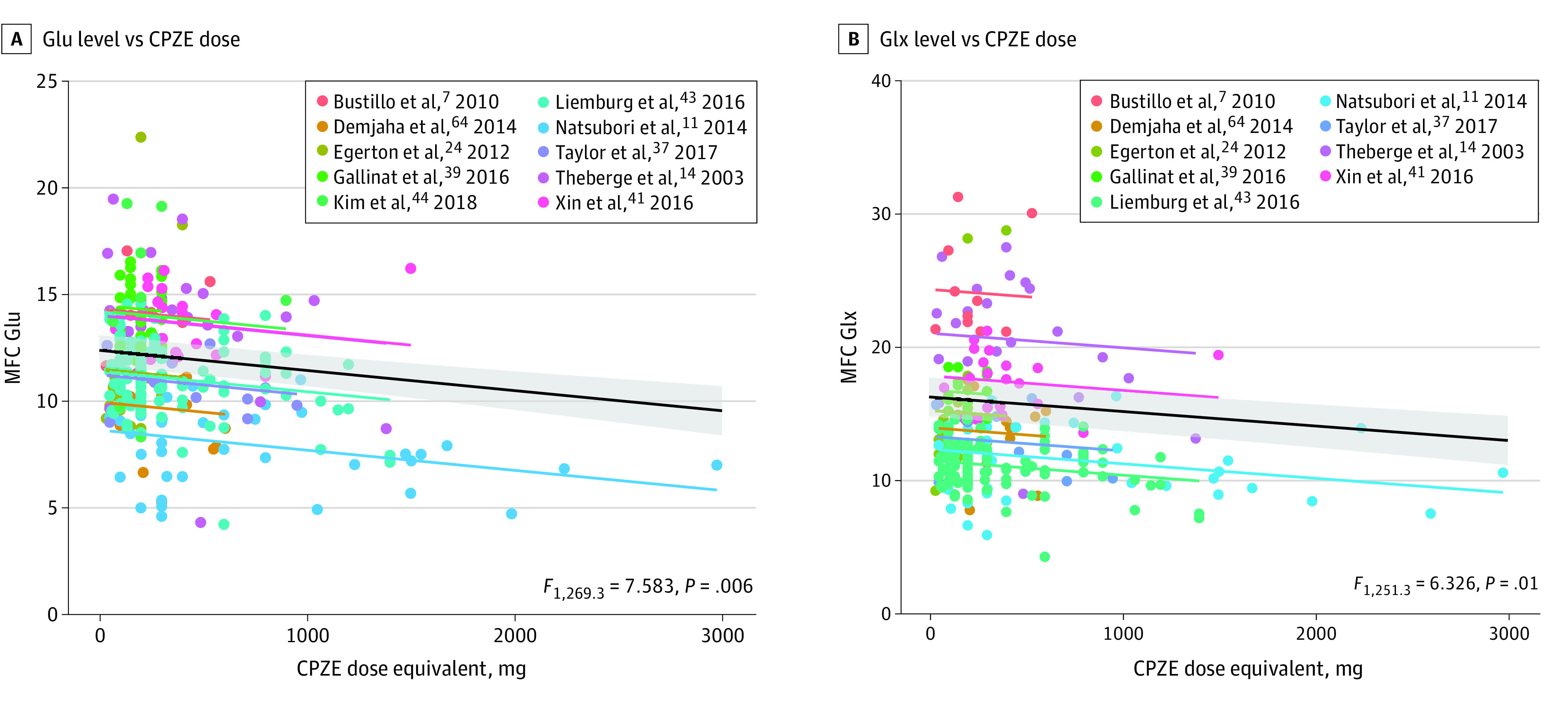
Correlations Between Chlorpromazine Equivalent (CPZE) Dose and Medial Frontal Cortex (MFC) Glutamatergic Metabolites A, Cerebrospinal fluid (CSF)–corrected glutamate (Glu) levels.^7,11,14,24,38,40,42,44,45,65^ B, CSF-corrected Glu plus glutamine (Glx) levels.^7,11,14,24,38,40,42,44,65^ The black line represents the linear mixed model, with SE represented by the gray shaded areas; the random-intercept models for each study listed are shown in different colors.

Both the MFC CSF-corrected Glu and CSF-corrected Glx levels were negatively associated with CPZE dose (CSF-corrected Glu level: *F*_1,269.3_ = 7.583, *P* = .006, n = 276; CSF-corrected Glx level: *F*_1,251.3_ = 6.326, *P* = .01, n = 259) (Figure 2). The CSF-corrected Glu level decreased by 0.10 per 100 mg of the CPZE dose (SE = 0.03), and the CSF-corrected Glx level decreased by 0.11 per 100 mg of the CPZE dose (SE = 0.04). The associations of the CPZE dose with the Glu to Cr and Glx to Cr ratios were nonsignificant.

When assessing the association of age with CPZE dose in the same model, the model combining age and CPZE dose best estimated the MFC CSF-corrected Glu level (Table 1). In contrast to the MFC, in the MTL, the Glx to Cr ratio was not significantly associated with age (n = 143 patients with schizophrenia, n = 151 healthy volunteers) or with CPZE dose (n = 94). There were insufficient data to examine the Glu to Cr ratio, the CSF-corrected Glu level, or the CSF-corrected Glx level in the MTL.

### Associations With Group

Both MFC CSF-corrected Glu and CSF-corrected Glx levels were lower in the schizophrenia group compared with the healthy volunteer group while controlling for age (*F*_1,1211.9_ = 4.311, *P* = .04, n = 596 healthy volunteers, n = 630 patients with schizophrenia; *F*_1,1079.2_ = 5.287, *P* = .02, n = 519 healthy volunteers, n = 573 patients with schizophrenia) (Table 1). There was no association of group with MFC Glu to Cr ratio or Glx to Cr ratio. However, although not statistically significant, MFC Cr levels were lower in patients compared with healthy volunteers while controlling for age (*F*_1,1395.9_ = 3.622, *P* = .06, n = 712 healthy volunteers, n = 705 patients with schizophrenia). In the MTL, the Cr level and the Glx to Cr ratio did not differ between patients and healthy volunteers.

### Association Between Glutamatergic Metabolite Levels and Symptom Severity

The PANSS total, positive, general, and negative subscores were all intercorrelated (eTable 2 in the Supplement); therefore, the initial model examined the PANSS total score. When significant, follow-up analyses investigated the PANSS positive score and the PANSS negative score in 1 model.

**Figure 3.**
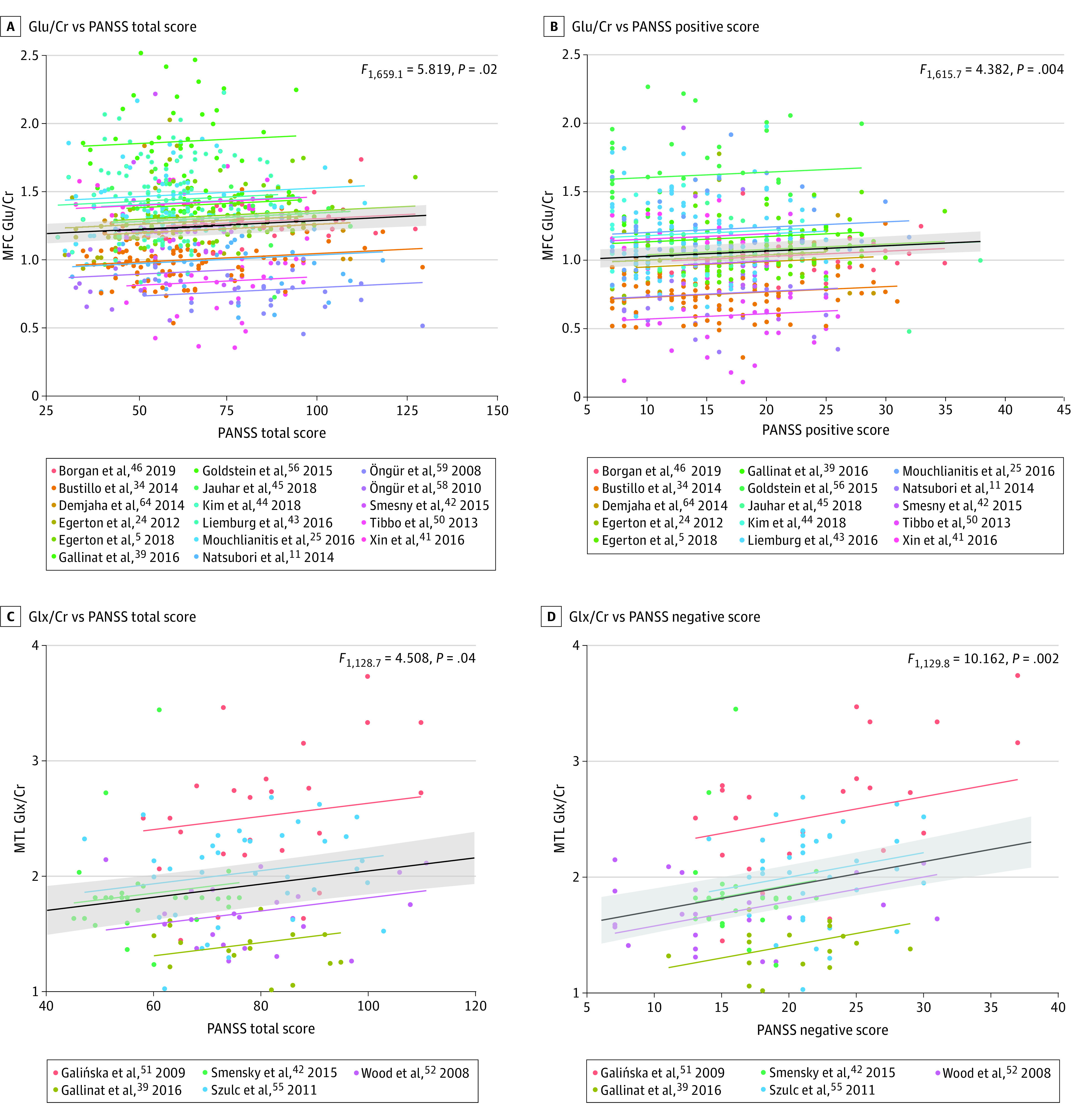
Correlations Between Medial Frontal Cortex (MFC) and Medial Temporal Lobe (MTL) Glutamatergic Metabolites and Positive and Negative Syndrome Scale (PANSS) Scores A, Positive association between the MFC glutamate to total creatinine plus phosphocreatine ratio (Glu/Cr) and the PANSS total score.^5,11,24,25,35,40,42,43,44,45,46,47,51,57,59,60,65^ B, PANSS positive score.^5,11,24,25,35,40,42,43,44,45,46,47,51,57,65^ C, Positive association between the MTL glutamate plus glutamine to Cr ratio (Glx/Cr) and PANSS total score. D, PANSS negative score.^40,43,52,53,56^ The black line represents the linear mixed model with SE represented by the gray shaded areas; the random-intercept models for each study listed are shown in different colors.

The MFC Glu to Cr ratio was positively associated with the PANSS total score (*F*_1,659.1_ = 5.819, *P* = .02, n = 668) (Figure 3). The Glu to Cr ratio increased by 0.01 per 10 points on the PANSS scale (SE = 0.005). Subsequent analysis found a positive association between the Glu to Cr ratio and the PANSS positive score (*F*_1,615.7_ = 4.382, *P* = .004, n = 625), whereby the Glu to Cr ratio increased by 0.04 per 10 points (SE = 0.02). The PANSS negative score was nonsignificant. The MFC Glu to Cr ratio was negatively associated with the GAF score (*F*_1,171.8_ = 13.152, *P* < .001, n = 178) (Figure 3), such that the Glu to Cr ratio increased by 0.04 per 10-point reduction on the GAF scale (SE = 0.01). There were no associations of CSF-corrected Glu or Glx level with the PANSS total or GAF score (Table 2).

**Table 2.** Associations of Measures of Symptom Severity and Social and Occupational Functioning With Glutamatergic Metabolites and Total Creatine and Phosphocreatine Levels

Brain region and source	No.	Glutamatergic metabolite, estimated mean (SE)	Clinical variable, mean (SD)	Statistics	Estimate (SE)
Medial frontal cortex					
Cr					
14 Studies^5,11,24,35,40,42,43,44,45,46,47,51,54,65^	559	9.21 (0.55)	PANSS total: 65.90 (18.36)	*F*_1,548.9_ = 0.365, *P* = .55	NA
6 Studies^5,24,40,46,51,65^	169	9.06 (0.42)	GAF: 49.76 (12.50)	*F*_1,164.2_ = 2.013, *P* = .16	NA
Glutamate, Cr-scaled					
17 Studies^5,11,24,25,35,40,42,43,44,45,46,47^ ^51,57,59,60,65^	668	1.25 (0.07)	Model 1: PANSS total: 65.44 (18.90) Model 2: age + PANSS Total^a^	*F*_1,659.1_ = 5.819, *P* = .02 *F*_1,661.5_ = 14.960, *P* < .001 *F*_1,659.3_ = 4.735, *P* = .03	0.0012 (0.0005) −0.0036 (0.0009) 0.0011 (0.0005)
15 Studies^5,11,24,25,35,40,42,43,44,45,46,47,51,57,65^	625	1.30 (0.06)	PANSS positive: 16.15 (6.04) PANSS negative: 16.87 (6.30)	*F*_1,615.7_ = 4.382, *P* = .004 *F*_1,614.3_ = 0.478, *P* = .49	0.0035 (0.0017) NA
6 Studies^5,24,40,46,51,65^	178	1.23 (0.09)	GAF: 50.04 (12.85)	*F*_1,171.8_ = 13.152, *P* = .001	−0.0041 (0.0011)
Glutamate, CSF-corrected					
12 Studies^5,11,24,35,40,42,43,44,45,46,47,65^	527	11.90 (0.62)	PANSS total: 65.46 (18.49)	*F*_1,520.8_ = 2.231, *P* = .14	NA
5 Studies^5,24,40,46,65^	140	11.55 (0.72)	GAF: 50.57 (12.81)	*F*_1,135.1_ = 2.043, *P* = .16	NA
Glx, Cr-scaled					
15 Studies^5,11,24,35,40,42,43,44,46^ ^47,49,57,59,65^	581	1.60 (0.09)	PANSS total: 64.97 (18.26)	*F*_1,571.3_ = 0.487, *P* = .48	NA
6 Studies^5,24,40,46,49,65^	155	1.56 (0.17)	GAF: 48.93 (14.07)	*F*_1,149.6_ = 1.720, *P* = .19	NA
Glx, CSF-corrected					
11 Studies^5,11,24,35,40,42,43,44,46,47,65^	497	15.48 (0.86)	PANSS total: 65.99 (18.54)	*F*_1,492.4_ = 0.227, *P* = .63	NA
5 Studies^5,24,40,46,65^	131	15.30 (0.87)	GAF: 50.53 (13.05)	*F*_1,128.1_ = 0.373, *P* = .54	NA
Medial temporal lobe					
Cr					
4 Studies^40,43,52,53^	109	5.03 (2.03)	PANSS total: 71.79 (15.46)	*F*_1,104.1_ = 0.797, *P* = .37	NA
Glx, Cr-scaled					
5 Studies^40,43,52,53,56^	132	1.90 (0.19)	PANSS total: 73.94 (15.50) PANSS positive: 17.42 (5.08) PANSS negative: 19.28 (5.86)	*F*_1,128.7_ = 4.508, *P* = .04 *F*_1,129.7_ = 0.000, *P* = .98 *F*_1,129.8_ = 10.162, *P* = .002	0.0057 (0.0027) −0.0212 (0.0067)
3 Studies^40,52,56^	76	1.97 (0.34)	CGI: 4.30 (0.98)	*F*_1,73.0_ = 10.914, *P* = .002	0.1976 (0.0598)

Abbreviations: AIC, Akaike information criterion; CGI, Clinical Global Impression; Cr, creatine plus phosphocreatine; GAF, Global Assessment of Functioning; Glu, glutamate; Glx, glutamate plus glutamine; NA, not applicable; PANSS, Positive and Negative Syndrome Scale.

^a^ Age and PANSS total score are both significantly associated with the medial frontal cortex Glu to Cr ratio, so we compared whether variance in the Glu to Cr ratio was best explained by model 1 including PANSS total score or model 2 including both PANSS total score and age (linear mixed methods estimated with maximum likelihoods). Model 2 showed the best fit (AIC, −98.7; residual deviance, −108.7) compared with model 1 (AIC, −85.9; residual deviance, −93.9) (*P* <. 001, determined by use of the χ^2^ test).

The MTL Glx to Cr ratio was positively associated with the PANSS total score (*F*_1,128.7_ = 4.508, *P* = .04, n = 132) (Figure 3). The Glx to Cr ratio increased by 0.06 per 10 points (SE = 0.03). Subsequent analysis found a positive association between the Glx to Cr ratio and the PANSS negative score (*F*_1,129.8_ = 10.162, *P* = .002, n = 132), such that the ratio increased by 0.2 per 10 points (SE = 0.07). No significant association was found for the PANSS positive score. The GAF data were unavailable. A higher Glx to Cr ratio was associated with a worse CGI score (*F*_1,73.0_ = 10.914, *P* = .002, n = 76), whereby the ratio increased by 0.2 per point on the CGI scale (SE = 0.06).

In the MFC, the PANSS total score was negatively associated with age, such that younger patients had more severe symptoms (eTable 2 in the Supplement). We compared whether the variance in the Glu to Cr ratio was best explained by age, PANSS total score, or both. The model including both age and PANSS total score showed the best fit (Table 2). The PANSS total score and CPZE dose were positively associated in the mega-analysis sample, such that patients with more severe symptoms received a higher CPZE dose (eTable 2 in the Supplement).

## Discussion

We conducted a participant-level mega-analysis to assess the association between 1H-MRS glutamatergic metabolite levels and the clinical and demographic features of schizophrenia. The main findings were negative associations of glutamatergic metabolite levels in the MFC with age in both patients with schizophrenia and healthy individuals and with the dose of antipsychotic medication in patients. Higher MFC Glu to Cr ratios were associated with more severe total and positive symptoms and with a lower level of overall functioning. In the MTL, elevated Glx to Cr ratios were associated with more severe total and negative symptoms and with worse CGI scores. In patients, MFC Glu levels were lower than in healthy volunteers irrespective of age, and there was a nonsignificant trend for lower Cr levels in patients. In the MFC, Cr levels, a measure commonly thought to be independent of age, increased with age in both patients and healthy volunteers. Overall, these results indicate that higher Glu levels may be associated with greater illness severity but that Glu levels may be reduced through effective antipsychotic treatment to below those observed in healthy volunteers.

The finding that MFC Glu levels decrease with age in patients with schizophrenia in a manner similar to healthy volunteers suggests that these reductions may reflect normal aging processes in this brain region (2%-3% reduction of mean Glu metabolite per decade). This is consistent with a recent meta-analysis of brain Glu metabolite levels in normal aging, which reports a larger effect size for Glu than Glx for age, as glutamine (part of the Glx signal) increases with age.^71^ In contrast to that meta-analysis, our mega-analysis did not detect changes associated with age in the MTL. This inconsistency may be due to the smaller number of studies available in this brain region, limiting our analysis to Glx levels and precluding assessment of Glu levels. One previous metaregression observed an accelerated effect of aging in patients compared with healthy volunteers^20^; however, this association may have been caused by a group of patients at ultra-high risk. Therefore, reduced Glu levels in patients compared with healthy volunteers in previous reports may not have been caused by greater age-related decline.

Conversely, MFC Cr levels increased with age in both patients and healthy volunteers (2% increase of mean Cr level per decade), consistent with previous studies,^72,73,74,75^ although 1 study reports no association.^76^ Creatine and phosphocreatine are involved in energy metabolism, and increased levels may reflect more burden on this system or increased glial cell numbers and activation with age.^77,78^ Caution should be taken when using Cr as a reference metabolite in the MFC because there was a trend for lower levels in patients. This lower level may have masked Glu differences between cases and controls, and thus lower patient Glu levels were detected only for CSF-corrected metabolites. Our findings are consistent with a report that the anterior cingulate cortex Cr level is negatively associated with schizophrenia spectrum liability.^79^ Therefore, future studies should prioritize CSF-corrected measures.

Our finding of lower MFC glutamatergic metabolite levels in patients with schizophrenia relative to healthy volunteers is consistent with a recent meta-analysis.^4^ Our study indicates that age and antipsychotic medication were independently associated with MFC Glu levels because the model incorporating both of these uncorrelated measures showed the best fit. This result suggests that findings of reduced Glu levels in patients compared with healthy volunteers are not associated with accelerated aging in patients but may be explained by greater antipsychotic exposure, although lower Glu levels have been reported in minimally treated patients with first-episode psychosis.^80,81^ This finding may explain reports of reduced anterior cingulate cortex Glu levels in patients with chronic schizophrenia compared with healthy volunteers.^9,10,11,12,13,14^ Indeed, a large longitudinal study reports a decrease in Cr-scaled Glu levels with treatment.^5^ Antipsychotic medication may reduce Glu levels indirectly, secondary to a reduction in dopaminergic signaling via striatal-cortical feedback loops.^82^ Studies indicate that this result is not necessarily associated with symptom improvement^5,49,83^ and that Glu levels remain elevated in patients nonresponsive to treatment, despite higher or similar doses of medication.^5,24,25,65,84,85,86^

Our third finding was that higher glutamatergic metabolite levels in both the MFC and MTL were associated with more severe symptoms and lower functioning. In the sample, younger patients were more likely to have severe symptoms, and the model incorporating both age and symptoms provided the best fit for the Glu data. Patients with more severe symptoms received a higher CPZE dose; thus, the association of symptoms with Glu level is not better explained by medication exposure. When symptom dimensions were subsequently examined, Glu metabolite levels in the MFC were associated with positive symptoms, whereas those in the MTL were associated with negative symptoms. The MFC and MTL are key brain regions implicated in schizophrenia. Glutamatergic outputs from these regions regulate dopamine release in the striatum, and excess dopamine release may underlie the development of psychotic symptoms.^87^ Hippocampal Glu level alterations may also be associated with learning and memory,^88^ relevant to negative symptoms. Associations with symptoms were observed for Cr-scaled but not CSF-corrected values. This association appears unlikely to be caused by creatine because creatine level was not associated with symptom severity.

### Strengths and Limitations

The strengths of the present study include the large patient sample (more than 700 patients), which enabled linear mixed models to account for potential collinearity. Mega-analyses are reported to be more sensitive than meta-analyses owing to narrower confidence intervals.^89^ Because data were assembled from different countries, the sample represents varying demographic features and clinical treatments.

The process of combining data from multiple independent sites also has limitations. The 1H-MRS acquisition protocols, MR imaging platforms, and scaling methods differed among studies, which we controlled for in the analysis by using linear mixed models to control for site effects and by separately considering CSF-corrected data from Cr-scaled data. Ideally, future prospective multicenter studies would further harmonize 1H-MRS acquisition and correction methods to enable more reliable data synthesis.^90^ Nevertheless, harmonization will always be constrained by the use of different MR imaging platforms across centers. Despite using established rating scales, there is a possibility of site effects associated with clinical assessment scores and CPZE dose calculations. Owing to a lack of data, we were unable to examine other brain regions that may be associated with schizophrenia pathophysiology. Therefore, we cannot determine whether the observed associations extend to other brain regions. The CPZE dose was not available for all studies; thus, analyses were restricted to smaller samples. Our analysis of the association between medication and Glu levels relied on cross-sectional data. Longitudinal studies can better examine the causal association between these factors, but our results are consistent with longitudinal studies reporting reduced MFC glutamatergic metabolite levels with treatment.^5,39,49,83,91,92^ Antipsychotic dose was associated with CSF-corrected Glu metabolite levels but not with Cr-scaled values. This finding contrasts with a large longitudinal 1H-MRS study finding a reduction in Cr-scaled Glu level with treatment.^5^ Finally, mega-analyses rely on contributed data, resulting in data omission.

## Conclusions

These findings have important implications for MRS studies in schizophrenia. They highlight the value of matching or adjusting for age, prioritizing CSF-corrected measures over Cr-scaled metabolite levels, and considering antipsychotic dose as an explanatory factor when comparing Glu levels between patients and healthy volunteers. The finding of elevated Glu levels in patients with more severe symptoms provides further support for the use of glutamatergic measures as a potential biomarker of illness severity, alongside other measures, and the development of novel treatments that target brain glutamatergic function.

## Notes

Supplement. eFigure. PRISMA Diagram eTable 1. Studies That Contributed Data eTable 2. Tests of Collinearity to Determine Which Variables Can Be Included in the Model
