# Supplementary material for: Association of Age, Antipsychotic Medication, and Symptom Severity in Schizophrenia With Proton Magnetic Resonance Spectroscopy Brain Glutamate Level: A Mega-analysis of Individual Participant-Level Data
Source: JAMA Psychiatry. 2021 Apr 21;78(6):1–16. doi: 10.1001/jamapsychiatry.2021.0380 (PMC8060889; doi:10.1001/jamapsychiatry.2021.0380)
Supplement: Supplement. — eFigure. PRISMA Diagram eTable 1. Studies That Contributed Data eTable 2. Tests of Collinearity to Determine Which Variables Can Be Included in the Model [file jamapsychiatry-e210380-s001.pdf]

Supplementary Online Content

Merritt K, McGuire PK, Egerton A, et al; 1H-MRS in Schizophrenia Investigators. Association of age, antipsychotic medication, and symptom severity in schizophrenia with proton magnetic resonance spectroscopy brain glutamate level: a mega-analysis of individual participant-level data. *JAMA Psychiatry*. Published online April 21, 2021. doi:10.1001/jamapsychiatry.2021.0380

eFigure. PRISMA Diagram

eTable 1. Studies That Contributed Data

eTable 2. Tests of Collinearity to Determine Which Variables Can Be Included in the Model

This supplementary material has been provided by the authors to give readers additional information about their work.

© 2021 Merritt K et al. *JAMA Psychiatry*.

eFigure. Prisma Diagram

The PRISMA IPD flow diagram

© Reproduced with permission of the PRISMA IPD Group, which encourages sharing and reuse for non commercial purposes

Identification

Number of studies identified through database searching (n = 1577)

Number of additional studies identified through other sources including contact with researchers

n = 18

Screening

Not in humans removed (n = 319) and not applicable studies removed (n = 934)

Eligibility

Number of studies screened for eligibility (n = 342)

Full-text articles excluded; not measure glutamate, glutamine or Glx (n = 129), review (n=38), overlapping samples (n = 31), Other = (n=30)

Obtaining data

Number of studies for which IPD were sought (n = 114)

Number of eligible Studies for which IPD were not sought (n = 7) in high risk cohorts

Available data

Number of studies for which IPD were provided (n = 45)

Number of participants for whom data were provided 1552 patients (including those at high risk) and 1221 healthy volunteers

Number participants for whom no data were provided (n = 0)

Number of studies for which IPD were not provided (n = 62) (no response)

Number of participants 1766 patients (including those at high risk) and 1453 healthy volunteers

Aggregate data not collected as previous meta-analyses have been conducted

Analysed data

IPD

Number of studies included in analysis (n = 42)

Number of participants included in analysis 1251 patients with schizophrenia and 1197 healthy volunteers.

Number participants excluded n = 301 high risk subjects and 24 healthy volunteers (J-resolved MRS data)

© 2021 Merritt K et al. *JAMA Psychiatry*.

eTable 1. Studies That Contributed Data

					MFC					MTL		Clinical data	
Study	Year	FEP	Chronic	Healthy Volunteer	Cr	Glu CSF	Glu/Cr	Glx CSF	Glx/Cr	Cr	Glx CSF	PANSS Total	CPZE
Block	2000	0	25	18	✗	✗	✗	✗	✗	✗	✗	✗	✗
Bloemen	2011	0	0	11	✓	✗	✓	✗	✓	✓	✗	✗	✗
Borgan	2019	28	0	33	✓	✓	✓	✓	✓	✗	✗	✓	✗
Bustillo	2010	15	0	12	✓	✓	✓	✓	✓	✗	✗	✗	✓
Bustillo	2014	17	78	89	✓	✓	✓	✓	✓	✗	✗	✓	✗
Capizzano	2011	0	0	25	✓	✗	✗	✗	✓	✓	✗	✗	✗
Coughlin	2015	7	18	17	✓	✗	✗	✗	✓	✗	✗	✗	✗
Demjaha	2014	0	14	10	✓	✓	✓	✓	✓	✗	✗	✓	✓
Dempster	2020	26	0	27	✓	✓	✓	✗	✗	✗	✗	✗	✗
Egerton	2012	32	0	0	✓	✓	✓	✓	✓	✗	✗	✓	✓
Egerton	2014	0	0	59	✓	✓	✓	✓	✓	✗	✗	✓	✗
Egerton	2018	72	0	60	✓	✓	✓	✓	✓	✗	✗	✓	✗
Fuente	2016	0	0	24	✓	✗	✗	✓	✗	✗	✗	✗	✗
Galinska	2009	30	0	19	✗	✗	✗	✗	✗	✓	✗	✓	✓
Gallinat	2016	16	13	29	✓	✓	✓	✓	✓	✓	✓	✓	✓
Goldstein	2015	0	48	18	✗	✗	✓	✗	✓	✗	✗	✓	✓
Goto	2012	18	0	18	✗	✗	✗	✗	✓	✗	✗	✓	✓
Jauhar	2018	26	0	20	✓	✓	✓	✓	✓	✗	✗	✓	✗
Kegeles	2000	0	10	10	✗	✗	✗	✗	✗	✗	✗	✗	✗
Kegeles	2012	0	32	22	✗	✗	✗	✗	✓	✗	✗	✗	✗
Kim	2018	19	0	49	✓	✓	✓	✗	✗	✗	✗	✓	✓
Liemburg	2016	37	110	36	✓	✓	✓	✓	✓	✗	✗	✓	✓
Mouchlianitis	2016	0	37	0	✗	✗	✓	✗	✗	✗	✗	✓	✗
Natsubori	2014	19	25	73	✓	✓	✓	✓	✓	✗	✗	✓	✓
Ongur	2008	0	27	37	✗	✗	✓	✗	✗	✗	✗	✓	✓
Ongur	2010	0	20	17	✓	✗	✓	✗	✓	✗	✗	✓	✗

© 2021 Merritt K et al. *JAMA Psychiatry*.

					MFC					MTL		Clinical data	
Ota	2015	0	17	0	✗	✗	✗	✗	✗	✗	✗	✓	✓
Plitman	2016	64	0	63	✗	✗	✗	✗	✗	✗	✗	✓	✗
Posporelis	2017	20	0	20	✓	✗	✓	✗	✓	✗	✗	✗	✓
Smesny	2015	31	0	31	✓	✓	✓	✓	✓	✓	✓	✓	✗
Stanley	1996	25	12	25	✗	✗	✗	✗	✗	✗	✗	✗	✓
Stanley	2007	18	0	61	✗	✗	✗	✗	✗	✗	✗	✗	✗
Stone	2009	0	0	27	✗	✗	✗	✗	✗	✓	✓	✓	✗
Szulc	2011	0	43	26	✗	✗	✗	✗	✗	✗	✗	✓	✓
Taylor	2017	11	5	18	✓	✓	✓	✓	✓	✗	✗	✗	✓
Theberge	2002	21	0	21	✓	✓	✓	✓	✓	✗	✗	✗	✗
Theberge	2003	0	21	21	✓	✓	✓	✓	✓	✗	✗	✗	✓
Tibbo	2013	33	0	41	✓	✗	✓	✗	✗	✗	✗	✓	✗
Wood	2007	0	15	14	✓	✗	✗	✗	✓	✗	✗	✓	✗
Wood	2008	34	0	19	✗	✗	✗	✗	✗	✓	✓	✓	✓
Wood	2010	0	0	29	✗	✗	✗	✗	✗	✗	✓	✗	✗
Xin	2016	25	0	33	✓	✓	✓	✓	✓	✗	✗	✓	✓
Yamasue	2003	0	16	15	✗	✗	✗	✗	✗	✗	✗	✓	✓

MFC; medial frontal cortex, MTL; medial temporal cortex, Cr; total creatine + phosphocreatine, Glu CSF; glutamate CSF-corrected, FEP; first-episode psychosis, PANSS; Positive and Negative Syndrome Scale, CPZE; Chlorpromazine equivalent dose.

© 2021 Merritt K et al. *JAMA Psychiatry*.

eTable 2. Tests of Collinearity to Determine Which Variables Can Be Included in the Model

	Medial Frontal Cortex				Medial Temporal Lobe
	Glutamate Cr-scaled	Glutamate CSF corrected	Glx Cr-scaled	Glx CSF corrected	Glx Cr-scaled
Age x Duration of Illness	r = 0.753, P<0.001	r = 0.715, P<0.001	r = 0.729, P<0.001	r = 0.712, P<0.001	r = 0.672, P<0.001
Age x CPZ Equivalent Dose	NS	NS	NS	NS	NS
CPZ Equivalent Dose x Duration of Illness	r = 0.142, P=.009	NS	NS	NS	NS
PANSS Total x Age	r = -0.113, P=0.004	r = -0.134, P=0.002	r = -0.165, P<0.001	r = -0.153, P<0.001	NS
PANSS Total x CPZ Equivalent Dose	r = 0.142, P= 0.017	r = 0.231, P<0.001	r = 0.161, P=0.009	r = 0.212, P=0.002	NS
PANSS Total x PANSS Positive	r = 0.792, P<0.001	r = 0.796, P<0.001	r = 0.788, P<0.001	r = 0.798, P<0.001	r = 0.544, P<0.001
PANSS Total x PANSS Negative	r = 0.741, P<0.001	r = 0.757, P<0.001	r = 0.739, P<0.001	r = 0.761, P<0.001	r = 0.630, P<0.001
PANSS Total x PANSS General	r = 0.934, P<0.001	r = 0.938, P<0.001	r = 0.936, P<0.001	r = 0.939, P<0.001	r = 0.942, P<0.001
PANSS Positive x PANSS General	r = 0.682, P<0.001	r = 0.684, P<0.001	r = 0.686, P<0.001	r = 0.689, P<0.001	r = 0.390, P<0.001
PANSS Positive x PANSS Negative	r = 0.322, P<0.001	r = 0.353, P<0.001	r = 0.314, P<0.001	r = 0.366, P<0.001	r =-0.175, P=.044
PANSS Negative x PANSS General	r = 0.560, P<0.001	r = 0.585, P<0.001	r = 0.556, P<0.001	r = 0.589, P<0.001	r = 0.489, P<0.001

If r values show a strong correlation then only one variable should be included in the model.

© 2021 Merritt K et al. *JAMA Psychiatry*.
